# Evaluation of Polydimethylsiloxane (PDMS) as a Substrate for the Realization of Flexible/Wearable Antennas and Sensors

**DOI:** 10.3390/mi14040735

**Published:** 2023-03-26

**Authors:** Praveen Kumar Sharma, Jae-Young Chung

**Affiliations:** 1 Research Center for Electrical and Information Technology, Seoul National University of Science and Technology, Seoul 01811, Republic of Korea; 2 Department of Electrical and Information Engineering, Seoul National University of Science and Technology, Seoul 01811, Republic of Korea

**Keywords:** anisotropy, bi-resonator, PDMS, bending, temperature dependence

## Abstract

To demonstrate that the silicone-based polymer polydimethylsiloxane (PDMS) is suitable as a substrate for flexible/wearable antennae and sensors, an investigation of its various properties was carried out. The substrate was first developed in compliance with the requirements, and then its anisotropy was investigated using an experimental bi-resonator approach. This material exhibited modest but discernible anisotropy, with values of ~6.2/25 % for the dielectric constant and loss tangent, respectively. Its anisotropic behavior was confirmed by a parallel dielectric constant (*ε_par_*) ~2.717 and an evaluated perpendicular dielectric constant (*ε_perp_*) ~2.570—*ε_par_* > *ε_perp_* by 5.7%. Temperature affected PDMS’s dielectric properties. Lastly, the simultaneous impact of bending and anisotropy of the flexible substrate PDMS on the resonance properties of planar structures was also addressed, and these had diametrically opposed effects. PDMS appears to be a good contender as a substrate for flexible/wearable antennae and sensors based on all experimental evaluations conducted for this research.

## 1. Introduction

Flexible and wearable electronics have evolved significantly during the past few years. Flexible electronics’ remarkable mechanical qualities, such as bending, stretching, and twisting, make them promising for modern electronic devices to operate in real-world conformal and varied environmental operating circumstances. With the emergence of flexible electronics, flexible/wearable antennae and sensors [1,2,3] have piqued the interest of academicians and industry personnel worldwide. They have a lot of appealing features, such as delivering adequate performance under a variety of operating conditions, which makes them a good contender for next-generation wireless communication systems.

There are a lot of choices of flexible substrates for the development of flexible and wearable antennae and sensors available in the literature, including fabric-, polymer-, and paper-based substrates [4,5,6]. Due to their multiple advantages over rigid and fabric substrates, polymers have been increasingly popular as a substrate for the design of flexible/wearable antenna sensors in the last few years. Flexible devices require bending, stretching, and twisting, and rigid substrates do not perform well under these conditions. Fabric substrates, though, can be employed for flexible antenna sensor designs; however, they are more vulnerable to external factors, such as humidity, temperature changes, and so on, which have a negative impact on the antenna’s radiating characteristics. Therefore, researchers are continuously exploring new conducting and substrate materials for the design of flexible antennae.

The silicone-based polymer polydimethylsiloxane (PDMS) (C_2_H_6_OSi)_n_ was chosen as the polymer substrate in this research. In addition to flexible antennae and sensors, it can also be employed as a flexible substrate in microchips, thin membranes, hydrophobic antenna coating, and other applications [7,8,9]. PDMS possesses favorable attributes for its employment as a flexible substrate: it is chemically and thermally stable, convenient to implement, adhesive, and has small anisotropy and homogeneous qualities, in addition to flexibility, transparency, and water-resistance properties [10,11].

In this research, by examining its various features, the applicability of PDMS as a substrate for sensors and flexible/wearable antennae is substantiated. The direction-dependent dielectric properties of the PDMS are assessed using the bi-resonator approach (Section 3) after the development of the PDMS (Section 2). Section 4 illustrates how the estimated dielectric properties change with temperature. In Section 5, the effect of anisotropy and bending on the resonant properties of structures built with flexible substrates such as PDMS is demonstrated, and Section 6 concludes the paper.

## 2. Development of PDMS

**Figure 1:**
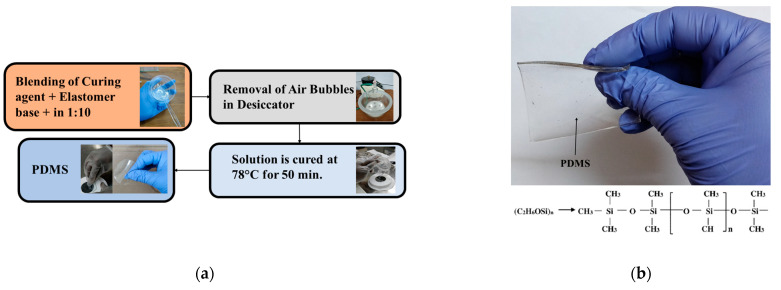
PDMS development process (**a**) and developed PDMS (**b**).

The physical and dielectric requirements for PDMS must be carefully considered in the development process, since each of these characteristics has a direct impact on the performance of the antennae and sensors. In order to formulate PDMS [11], as shown in Figure 1, a silicone curing agent and base must first be completely blended in a clean vessel in a circular motion at a ratio of 1:10. The solution is then placed in the desiccator until all air bubbles that form during mixing have been eliminated. The thickness of Teflon boats can be chosen according to the requirements of PDMS thickness. The liquid PDMS solution is then poured onto an incredibly thin circular silicon wafer that has been placed on the boat. Following that, this solution is cured for about 50 min at a temperature of 78 °C. Finally, the transparent layer of PDMS is gently scraped off for its application in sensors and antennae after considerable characterization.

## 3. Investigation of Direction-Specific Dielectric Parameters (Anisotropy) of PDMS: Bi-Resonator Method

Specifications of the materials, which include the dielectric values and information on the characterization techniques utilized, are provided by the various substrate producers. These values are suitable for isotropic substrates (which have the same dielectric values in all directions); however, many contemporary substrates are reinforced, which means they are combined with other materials, such as fabric substrates, etc. Since their dielectric characteristics can be changed to exhibit anisotropic behavior, the given values are not applicable to their use in sensors and antennae. It has been deduced from the literature that various studies have employed variable values for the loss tangents, tanδ*_r_* and dielectric constants, *ε_r_* of PDMS, which are in the range of 2.3–3.0 for *ε_r_* (1–6 GHz) [12,13,14]. These variances are a result of different characterization techniques used, such as the Kent and Courtney methods, which provide values of the dielectric properties parallel to the substrate surface, and TM-650 and reentrant methods measure the perpendicular dielectric properties [15,16,17,18]. These techniques produce a variety of outcomes when used on anisotropic substrates, and not all of them are appropriate for PDMS characterization.

Consequently, a comprehensive methodology that can be employed on anisotropic polymer substrates is required. It is possible to determine important details about the interior structures and compositions of anisotropic substrates from their characterization, which aids in determining their suitability for different applications. In this research, PDMS is characterized using a bi-resonator experimental technique. This approach uses two cylindrical resonators to conduct measurements of the dielectric properties in parallel and perpendicular directions and confirms its anisotropic behavior.

### Bi-Resonator Method

**Figure 2:**
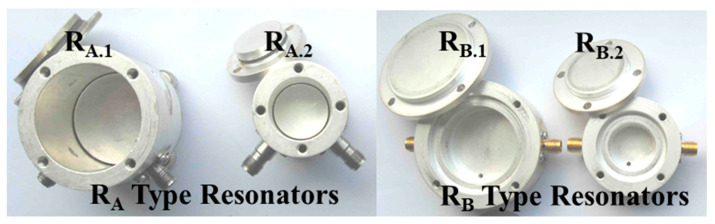
R_A_- and R_B_-type resonators (the diameters- *D_A.1_* = 30.5 mm, *D_A.2_* = 18.2 mm, *D_B._*_1_ = 30.5 mm, *D_B.2_* = 18.3 mm).

This method was utilized to investigate the direction-dependent dielectric characteristics of PDMS. A bi-resonator consists of two cylindrical resonators, R_A_ and R_B_, as the name would imply, and is shown in Figure 2. For the estimation of the perpendicular dielectric parameters, the R_B_ is designed to support the TM modes, whereas the R_A_ is meant to support the TE modes for the determination of the parallel dielectric parameters. In R_A_, the sample is positioned in the middle (exactly at its half), as the electric field is oriented along the surface and is strongest at half the resonator’s height. In order to distinguish the applied mode (TE_011_) from other lower-order and higher-order modes, the resonator’s diameter (*D_A_*) is taken to be approximately equal to its height (*H_A_*) while conducting the measurements. The R_B_ resonator is made to accommodate the TM_010_ mode, in which the electric field is perpendicular to the surface of the material being tested. In this mode, too, the sample is positioned at the bottom of the resonator to distinguish it from the other modes. Here, it is assumed that the resonator’s height is less than half of its diameter (*D_B_*). The extraction procedure using both of the resonators depends on the s_d_. There are two possibilities if the diameter of the sample *s_d_ ~ D_A_, D_B_* the analytical procedure can be applied [19], but if the *s_d_ < D_A_, D_B_* the investigation of dielectric values of PDMS is implemented by electromagnetic simulations due to the increased complexity of the analytical approach.

The steps followed throughout the measurement process are first, the empty resonators are measured to determine the resonance frequencies (*f_e1,2_*) and unloaded quality factors (*Q_e1,2_*) of the chosen mode in order to determine the equivalent diameter (*D_eqv1,2_*) and wall conductivity (*σ_eqv1,2_*) (Table 1). The idea behind utilizing the *D_eqv_* is that when resonance parameters are simulated and measured for resonators with fixed dimensions, they do not quite match. There are numerous causes for this, including temperature changes, coupling probe influence, tuning screws, and dimension uncertainties. Therefore, comparable parameters are employed to solve this issue. When a coincidence between the simulation and the measurement is attained, the values of these parameters are adjusted in simulations. Next, the PDMS-containing resonators are assessed again for the *f_s1,2_* and *Q_s1,2_* values, using the relevant method, depending upon the diameter of the sample from which the *ε_par_*/*tanδ_par_* and *ε_perp_*/*tanδ_perp_* are extracted.

**Figure 3:**
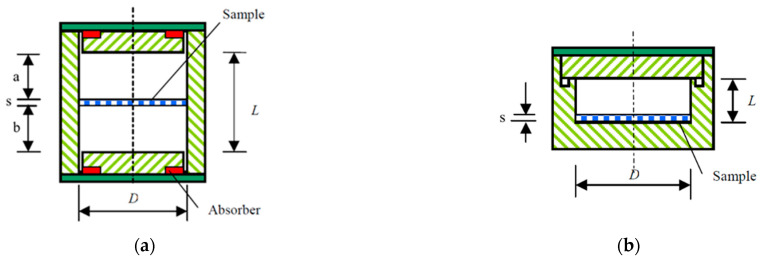
Representations of resonator R_A_ (**a**) and R_B_ (**b**) for the analytical approach when *s_d_ ~ D_A_, D_B_*.

There are two cases of the evaluation methods depending upon *s_d_* (diameter of the sample):

Case 1: If *s_d_ ~ D_A_, D_B_,* the analytical approach is followed [19] considering the given set of equations:

For parallel values, consider Figure 3a

(1) $$\frac{tan\beta_{s}s}{s}=\frac{(tan\beta_{e}a+tan\beta_{e}b)/\beta_{e}}{\left[\varepsilon_{par}+\left(\varepsilon_{par}-1\right){\left(\frac{\chi }{\beta_{e}}\right)}^{2}\right]tan\beta_{e}atan\beta_{e}b-1}$$

(2) $${\beta_{e}}^{2}={\left(2\pi /\lambda_{0}\right)}^{2}-{\chi_{mn}}^{2}$$

(3) $${\beta_{s}}^{2}={\varepsilon_{par}(2\pi /\lambda_{0})}^{2}-{\chi_{mn}}^{2}$$

(4) $$\chi_{mn}=2\upsilon_{mn}^{\prime }/D$$

(5) $$\upsilon_{01}^{\prime }=3.8317$$

(6) $$tan\delta_{par}=\frac{1}{\varepsilon_{par}}\left\{\frac{1}{Q_{s}}-\frac{1}{Q_{e}}\right\}{\left\{\frac{s}{L}-\frac{1}{\pi }sin\frac{\pi s}{L}\right\}}^{-1}$$

and Figure 3b for perpendicular values

(7) $$\varepsilon_{perp}=1+\frac{f_{e}-f_{s}}{f_{e}}{\left[\frac{s}{2L}-D_{e}\left(1-\frac{s}{L}\right)\frac{f_{e}-f_{s}}{f_{e}}\right]}^{-1}$$

(8) $$D_{pe}=D_{p}\frac{\pi s}{2L}cotan\frac{\pi s}{2L}$$

(9) $$D_{p}=\frac{1-s/D}{\surd (1+{\left(\frac{s}{D}\right)}^{2})}$$

(10) $$tan\delta_{perp}=\frac{\frac{1}{Q_{s}}-\frac{1}{Q_{e}}}{2\left[\frac{s}{2L}-D_{pe}\left(1-\frac{s}{L}\right)\frac{f_{e}-f_{s}}{f_{e}}\right][1+2\frac{f_{e}-f_{s}}{f_{e}}]}$$

where in the above equations *β_e_* and *β_s_* are empty and with the sample resonator’s propagation constant, respectively, and *χ_mn_* represents the eigenvalues that are determined through the roots of the Bessel function derivative for the particular mode. *D_pe_* is the effective depolarization factor that depends on the depolarization factor *D_p_*.

**Figure 4:**
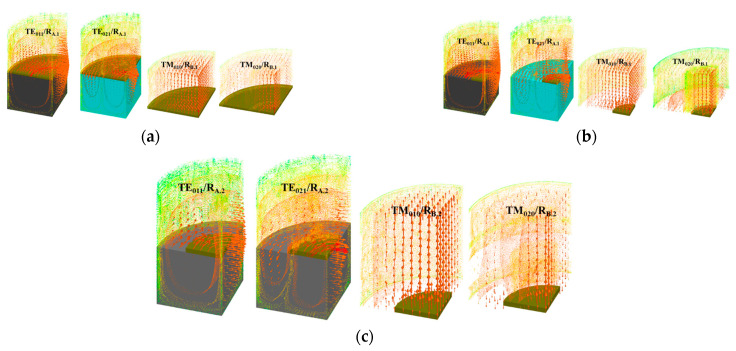
EM simulations of resonators R_A_ and R_B_: when *s_d_ ~ D_A_, D_B_* (**a**), *s_d_ < D_A_, D_B_* (**b**,**c**).

Case 2: If the *s_d_ < D_A_, D_B_*, the analytical method is inappropriate in this situation. Electromagnetic simulations can be used in this situation, as shown in Figure 4. The values of the dielectric parameter are adjusted until the simulations provide resonant frequencies and quality factors that correspond with measured values.

Table 2 lists the PDMS dielectric parameter values that have been extracted by the applied method. The data that are presented demonstrate that the PDMS has a modest but detectable anisotropy, as all of the results for both of the resonators are unique. Other similar materials are also characterized using this technique in order to validate the applied methodology, and all of the results are reported in Table 3 along with the averaged results for PDMS. The outcomes for different materials are quite decent. The following relations [20] are used to compute the anisotropy for each of these materials:

(11) $${∆Aniso}_{\varepsilon }=2[\frac{\left(\varepsilon_{par}-\varepsilon_{perp}\right)}{\left(\varepsilon_{par}+\varepsilon_{perp}\right)}]$$

(12) $${∆Aniso}_{tan\delta }=2[\frac{\left({tan\delta }_{par}-{tan\delta }_{perp}\right)}{\left({tan\delta }_{par}+{tan\delta }_{perp}\right)}]$$

**Figure 5:**
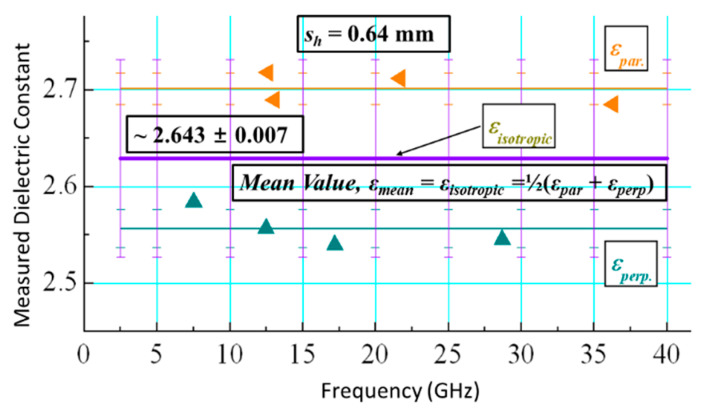
PDMS dielectric constant’s measured values.

The PDMS samples’ anisotropy is estimated to be 6.2%/25% by applying the above equations. The PDMS material’s chain structure and porous architecture are correlated with its anisotropy, which is further substantiated by the temperature measurements conducted in the later section of this paper. When used in antenna and sensor applications, the PDMS material can be considered almost isotropic by taking into account its isotropic equivalent values (mean), which lie between the parallel and perpendicular values, as illustrated in Figure 5. The mean values are *ε_mean_* = *ε_isotropic_* ~ 2.643 ± 0.007 and tan*δ_mean_* = tan*δ_isotropic_* ~ 0.0281 ± 0.0009 for 2.5–40 GHz.

## 4. Variation of the Dielectric Parameters of PDMS with Temperature

One of the novel experiments described in this research examines how temperature fluctuations affect the dielectric characteristics of PDMS. It is crucial to comprehend this phenomenon in order to use PDMS as a substrate in flexible antennae and sensors for the following reasons: (i) it is naturally anisotropic, (ii) the dielectric properties depend on the measurement frequencies, and (iii) PDMS has a significant thermal coefficient of expansion that ranges from three hundred at 149 °C from −55 °C [21].

**Figure 6:**
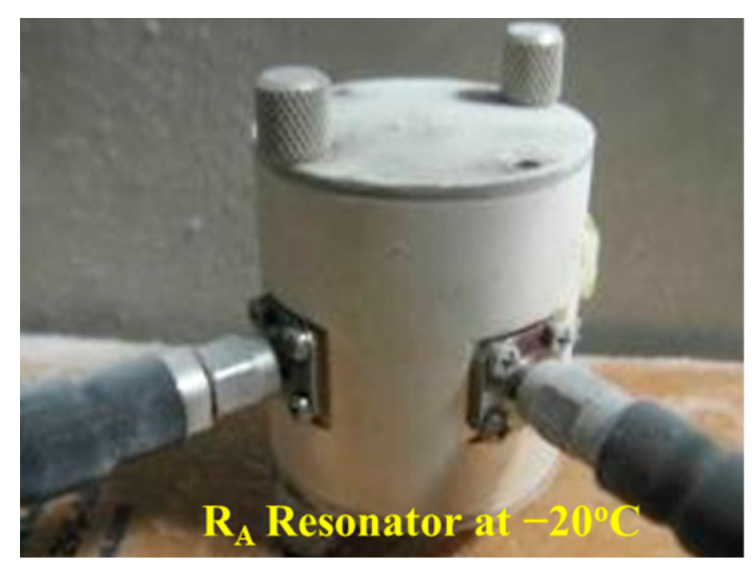
R_A_ resonator in the Thermotron chamber.

In commercially accessible thermal chambers such as Thermotrons (−40 °C to +110 °C: ±2 °C) as shown in Figure 6, the employed resonance process is repeated at various temperatures to ensure temperature stability. Each chamber undoubtedly has its own temperature gradient, but because the resonators are so small and the measurements are only taken for around 15 min, it is believed that the operating temperature is constant.

The measurements are conducted only for the first two resonators of types R_A_ and R_B_ using the identical order modes as before, because the measurement process is fairly drawn out and time-consuming. The measurement process is the same as well: initially, the empty resonators (*f*_e1,2_ and *Q*_e1,2_) are measured at the chosen temperatures. It is crucial to carry out the measurements at the equivalent values of the diameter and wall conductivities (*D_eqv_*_1,2_ and *σ_eqv_*_1,2_). This is because temperature variations, resonator wall expansion, and resistance changes affect the measured resonating frequencies and quality factor values for both resonators, and this also improves the accuracy of the measurement procedure used. The PDMS dielectric values are then examined in both perpendicular and parallel directions by measuring resonators with the samples at the same temperatures.

**Figure 7:**
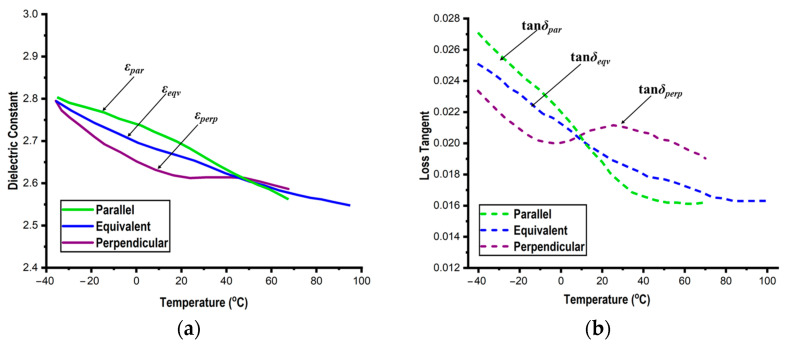
Dielectric parameters: dielectric constants (**a**) and loss tangents (**b**) of PDMS at various temperatures (5–15 GHz).

Table 4 shows the measured values for the empty resonators at various temperatures, and Table 5 shows the measured values for PDMS. These measurement values pertain to the three PDMS materials that were averaged. Additionally, Figure 7 shows the effect of variation in the PDMS dielectric characteristics (from 5 GHz to 15 GHz) with temperatures. As the temperature varies, the dielectric constants vary inversely in both directions, ranging from 2.57 to 2.79 as the temperature decreases, for instance.

As observed from Figure 7, between −25 °C and +30 °C is where the PDMS’s apparent anisotropy occurs. The loss tangents also exhibit highly precise temperature response: parallel values of loss tangents are smaller than perpendicular values at low temperatures and vice versa.

**Figure 8:**
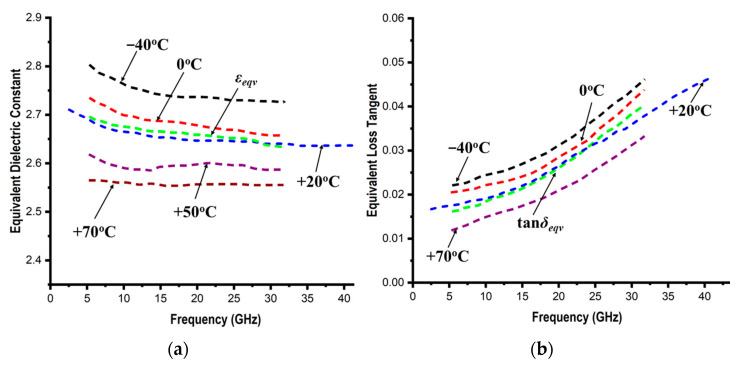
Variation in equivalent dielectric parameters: dielectric constants (**a**) and loss tangents (**b**) of PDMS at different temperatures with respect to the frequency.

Table 5 and Figure 8 present the extracted values, which are seen to be in good agreement with Figure 7 for both scenarios. Here too, the equivalent values rise as the temperature falls. Due to the material’s porous nature, which causes the air fraction to increase as the temperature rises while the polymer fraction decreases, this phenomenon occurs. This also explains the anisotropic nature of this material.

## 5. Bending and Anisotropy Influence

This section examines the impact of bending and anisotropy on the radiation properties of antennae and sensors employing flexible substrates, including PDMS. The implications must be carefully taken into account while designing flexible and wearable antennae and sensors. A majority of researchers argue that inaccurate measurement circumstances are to blame for the difference between simulated and experimental findings [22]. Quite a few researchers have cited the precise cause of it. The substrate’s height, anisotropy, and bending nature, which are crucial in this situation, are examined here.

**Figure 9:**
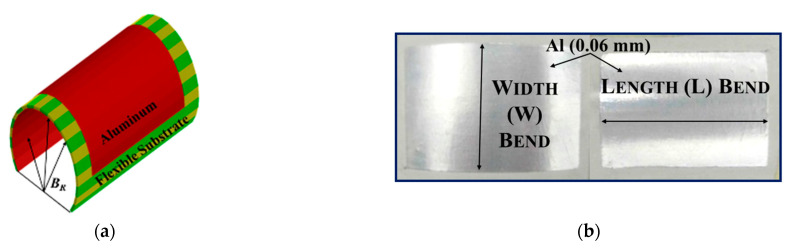
Bending Radius (**a**), Bending Scenarios (**b**).

**Figure 10:**
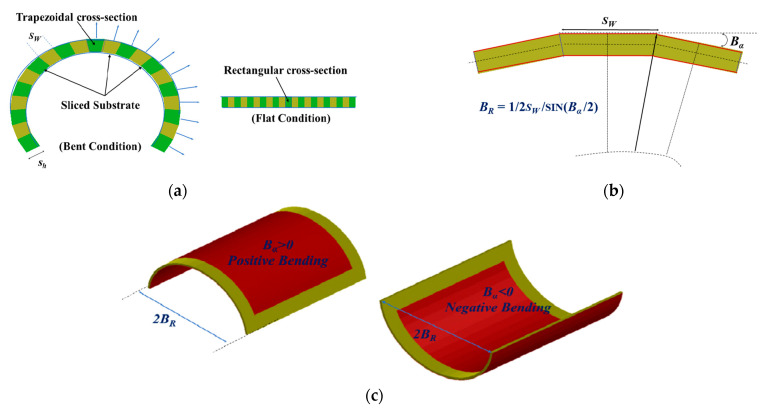
(**a**) Division of the flexible substrates into equal slices with a different cross sections in flat and bent conditions, (**b**) relation between bending angle and radius, and (**c**) orientation of bending with respect to the bending angle.

The bending radius *B_R_* (Figure 9a) to which the device is bent is typically used to assess the effect of bending. Two distinct bending scenarios—width and length bending—(Figure 9b) are chosen in order to examine the impact of bending on aluminum planar structures (with a thickness of 0.06 mm). Geometrical modeling is employed in simulations to investigate the effect of bending of flexible substrates. As shown in Figure 10a, the substrate is split into several equal pieces with rectangular contours for flat conditions and trapezoidal sections for bent conditions. To access the bending in this research, a new parameter *B_α_* (bending angle) is implemented, as shown in Figure 10b. Here, two different cases of bending with respect to the bending angle are considered: positive bending, where *B_α_* > 0, and negative bending, where *B_α_* < 0 (Figure 10c).

**Figure 11:**
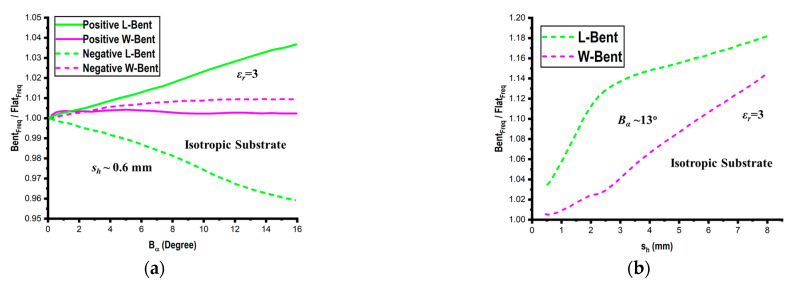
Bending effect on an isotropic substrate with respect to the bending angle (*B_α_*) (**a**) and substrate height (*s_h_*) (**b**).

First, bending measurements are conducted on the isotropic substrate (*ε_r_* = 3) in order to carry out the comparison. With respect to the bending angle (*B_α_*) and height (*s_h_*) of the substrate, Figure 11 illustrates the impact of bending utilizing an isotropic substrate on the resonant frequencies for both flat and bent structures. These graphs demonstrate that for isotropic substrates, length-bent structures have higher resonant frequencies than flat structures. For positive bending and vice versa for negative bending, the effect of the bending is reduced for width-bent structures. The bending has little influence because the lowest-order mode’s standing wave (TM_10_) is located exactly along the curvature of length-bent structures, while standing waves in width-bent structures are located in a perpendicular direction. This is due to the fact that the electric length of the entire structure on the substrate decreases and geometric length increases as *B_α_* increases.

**Figure 12:**
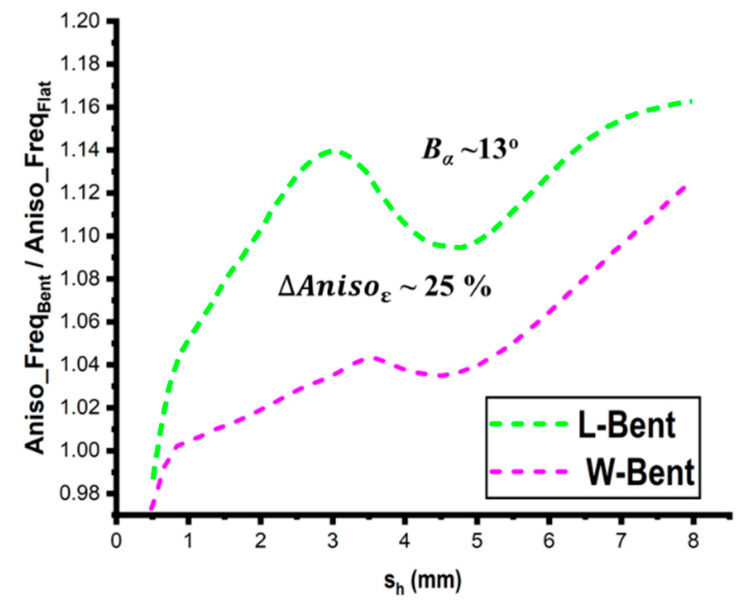
Bending effect on an anisotropic substrate with respect to the substrate height (*s_h_*).

Measurements have been conducted for flat and bend situations (*B_α_* ~ 13°) in order to study the effects of bending on the resonant properties of planar structures using anisotropic substrates (25%) with respect to the substrate height (*s_h_*), as shown in Figure 12. It is evident that on thicker substrates, the bending influence can balance out the anisotropy effect.

**Figure 13:**
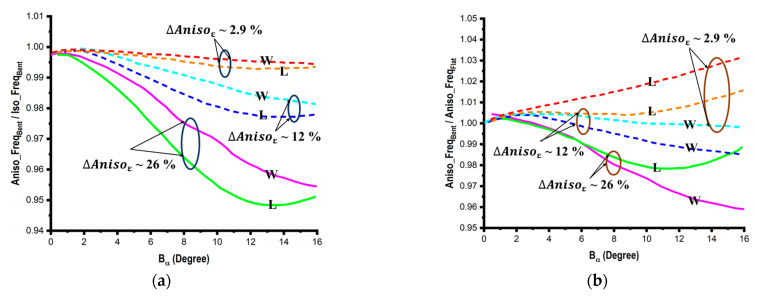
(**a**) Comparison of the effect of bending on the resonant frequencies of the isotropic and anisotropic substrates and (**b**) concurrent effects of bending and anisotropy.

**Figure 14:**
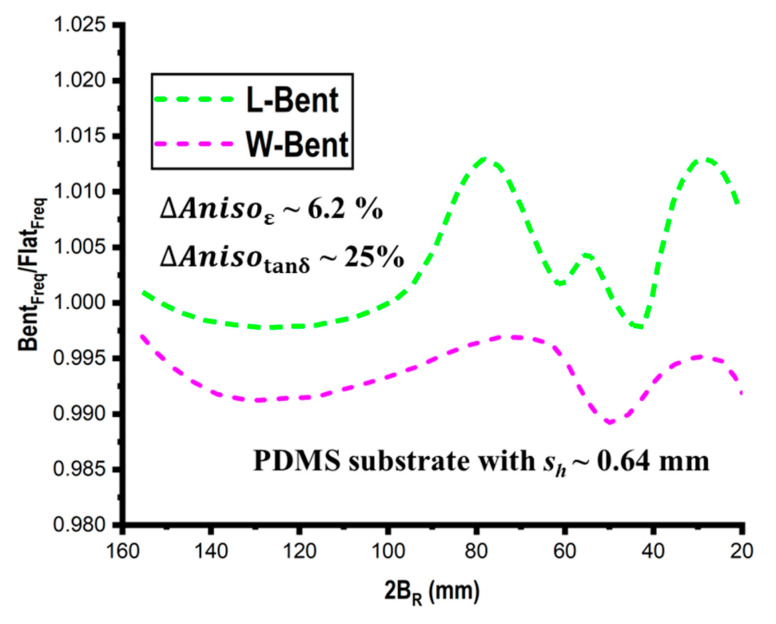
Effect of bending and anisotropy of the PDMS on its resonant characteristics.

Using simulation models, it is possible to examine how profoundly bending affects the resonant properties of various bent planar structures at various bending angles. First, considering the two examples of length (L) and width (W) bend as previously indicated, a comparison is conducted between the anisotropic and isotropic substrates, as illustrated in Figure 13. The anisotropic substrates are divided into three ranges: low (~2.9%), middle (~12%), and high (~26%). The obtained results show that the effect of anisotropy on bent structures is significantly greater than that on unbent structures, which is important information. The obtained results show that—in contrast to the common isotropic situation—anisotropy has a negative impact on resonating frequencies. Therefore, it can be inferred that bending and anisotropy’s effects are just the antithesis of one another. The resonant conditions for the flat and bending circumstances utilizing the rectangular structure (L_s_ = 36 mm and W_s_ = 25 mm) and the PDMS substrate with *s_h_* ~ 0.6 mm are depicted in Figure 14, which also depicts the resultant finding.

## 6. Conclusions

In this research, PDMS material characteristics were investigated experimentally to verify its viability as a flexible substrate for antenna and sensor applications. By employing the bi-resonator method, PDMS parallel and perpendicular dielectric characteristics (*ε_par_*/*tanδ_par_*, *ε_perp_/tanδ_perp_*) were estimated to be 2.717/0.0360 and 2.570/0.0203, respectively. Perpendicular values were ~5.7% lower than the parallel values, and the mean values fell between these obtained values (2.643/0.0281). Empirical evidence of the fluctuation of the PDMS dielectric parameters at various temperatures illustrated that both sets of parameters drop as the temperature rises: *tanδ_perp_* > *tanδ_par_* at low temperatures, and as the temperature rises, *tanδ_perp_* < *tanδ_par_*. This validates the anisotropy of PDMS and is predominantly driven by the change in polymer and air fractional volumes with temperature. Finally, the geometrical approach in simulations was used to examine the combined effects of bending and anisotropy on the resonance characteristics of structures using anisotropic flexible substrates. The substrate was split into multiple slices of equal size, and the bending angle *B_α_* was then employed as a parameter for the bending analysis. It was revealed that the cumulative effects of bending and anisotropy have contrary implications for the resonating characteristics.

## Figures and Tables

**Table 1:** Measured parameters for the empty resonators.

Resonator Type (R_A_/R_B_)	Diameter (*D_A/B_*) mm	Height (*H_A/B_*) mm	Typical Res. Frequency (f_e1,2_) of Used Modes GHz	Typical Unloaded Q Factor (*Q_e1,2_*) of Used Modes	Equivalent Diameter (*D_eqv1,2_*) mm	Wall Conductivity (*σ_eqv1,2_*) S/m
R_A.1_	30.50	30.50	13.1620/TE_011_ 19.2897/TE_013_ 21.8426/TE_021_ 32.5980/TE_031_	19,213 15,671 5101 4978	30.0642 30.0794 30.0489 30.0594	3.152 × 10^7^ 1.453 × 10^7^ 1.234 × 10^6^ 8.572 × 10^5^
R_A.2_	18.20	18.15	21.7990/TE_011_ 37.8514/TE_021_	13,281 2874	18.1361 18.1245	2.465 × 10^7^ 6.452 × 10^5^
R_B.1_	30.50	12.26	7.6559/TM_010_ 17.5305/TM_020_ 27.4883/TM_030_	6910 7632 9875	30.0234 30.0145 30.0253	3.589 × 10^7^ 1.845 × 10^7^ 1.975 × 10^7^
R_B.2_	18.30	12.20	12.6381/TM_010_ 29.0065/TM_020_	7280 5642	18.1448 18.1502	3.779 × 10^7^ 1.031 × 10^7^

**Table 2:** Extracted dielectric parameters of PDMS (**a**) parallel values, and perpendicular values (**b**).

**(a)**			
**PDMS Sample No. (Diameter, *s_d_* mm; Height, *s_h_* mm)**	**Parallel Dielectric Constant (** ***ε_par_*)**	**Parallel Loss Tangent (*tan*** ***δ_par_*)**	**Resonance Frequency *f_s_* (GHz)/Mode**
**Resonator R_A.1_ (*D_A.1_* = 30.50 mm; *H_A.1_* = 30.50 mm)**			
1 (30.50; 0.67 ± 0.09)	2.719 ± 0.006 2.715 ± 0.005	0.0362 ± 0.0009 0.0359 ± 0.0012	12.609/TE_011_ 21.675/TE_021_
2 (18.20; 0.71 ± 0.03)	2.663 ± 0.007 2.701 ± 0.009	0.0255 ± 0.0009 0.0290 ± 0.0017	12.786/TE_011_ 22.231/TE_021_
3 (14.9; 0.58 ± 0.06)	2.689 ± 0.007 2.710 ± 0.005	0.0241 ± 0.0001 0.0282 ± 0.0012	12.887/TE_011_ 22.285/TE_021_
4 (10.1; 0.59 ± 0.02)	2.597 ± 0.004 2.607 ± 0.003	0.0258 ± 0.0015 0.0261 ± 0.0009	13.058/TE_011_ 22.38/TE_021_
5 (7.8; 0.72 ± 0.05)	2.806 ± 0.014 2.781 ± 0.012	0.0275 ± 0.0014 0.0281 ± 0.0012	13.058/TE_011_ 22.751/TE_021_
**Resonator R_A.2_ (*D_A.2_* = 18.20 mm; *H_A.2_* = 18.15 mm)**			
1 (18.20; 0.71 ± 0.03)	2.712 ± 0.002 2.691 ± 0.005	0.0274 ± 0.0006 0.0280 ± 0.0005	20.402/TE_011_ 35.238/TE_021_
2 (10.1; 0.59 ± 0.02)	2.697 ± 0.005	0.0254 ± 0.0015	13.256/TE_011_
3 (7.8; 0.72 ± 0.05)	2.735 ± 0.012	0.0236 ± 0.0014	21.753/TE_011_
**(b)**			
**PDMS Sample No. (Diameter, *s_d_* mm; Height, *s_h_* mm)**	**Perpendicular Dielectric Constant (** ***ε_perp_*)**	**Perpendicular Loss Tangent (*tan*** ***δ_perp_*)**	**Resonance frequency *f_s_* (GHz)/mode**
**Resonator R_B.1_ (*D_B.1_* = 30.50 mm; *H_B.1_* = 12.26 mm)**			
1 (30.50; 0.67 ± 0.09)	2.590 ± 0.007 2.550 ± 0.014	0.0174 ± 0.0006 0.0232 ± 0.0013	7.520/TM_010_ 17.185/TM_020_
2 (18.20; 0.71 ± 0.03)	2.585 ± 0.009 2.508 ± 0.012	0.0201 ± 0.0004 0.0195 ± 0.0014	7.579/TM_010_ 17.391/TM_020_
3 (14.9; 0.58 ± 0.06)	2.589 ± 0.009 2.568 ± 0.010	0.0205 ± 0.0009 0.0249 ± 0.0009	7.578/TM_010_ 17.267/TM_020_
4 (10.1; 0.59 ± 0.02)	2.598 ± 0.005 2.567 ± 0.012	0.0186 ± 0.0008 0.0224 ± 0.0012	7.682/TM_010_ 17.425/TM_020_
5 (7.8; 0.72 ± 0.05)	2.585 ± 0.014 2.568 ± 0.012	0.0210 ± 0.0004 0.0220 ± 0.0016	7.612/TM_010_ 17.485/TM_020_
**Resonator R_B.2_ (*D_B.2_* = 18.30 mm; *H_B.2_* = 12.20 mm)**			
1 (18.20; 0.71 ± 0.03)	2.601 ± 0.004 2.572 ± 0.012	0.0232 ± 0.0007 0.0265 ± 0.0012	12.514/TM_010_ 28.981/TM_020_
2 (14.9; 0.58 ± 0.06)	2.490 ± 0.011 2.458 ± 0.016	0.0189 ± 0.0012 0.0211 ± 0.0011	12.589/TM_010_ 28.612/TM_020_
3 (10.1; 0.59 ± 0.02)	2.580 ± 0.012 2.498 ± 0.013	0.0188 ± 0.0012 0.0215 ± 0.0010	12.899/TM_010_ 29.8752/TM_020_
4 (7.8; 0.72 ± 0.05)	2.601 ± 0.018 2.632 ± 0.016	0.0235 ± 0.0014 0.0291 ± 0.0011	12.465/TM_010_ 29.152/TM_020_

**Table 3:** Comparison of PDMS dielectric parameters with other similar materials evaluated using the same method.

Substrates	*ε_par_*	*tanδ_par_*	*ε_perp_*	*tanδ_perp_*	*ε_eqv_*	*tanδ_eqv_*
Polydimethylsiloxane (PDMS)	2.717 ± 0.005	0.0360 ± 0.0010	2.570 ± 0.010	0.0203 ± 0.0009	2.643 ± 0.007	0.0281 ± 0.0009
Polytetrafluoroethylene (PTFE)	2.052 ± 0.007	0.00034 ± 0.00011	2.035 ± 0.018	0.00021 ± 0.00004	2.043 ± 0.012	0.00027 ± 0.00007
Cyclic olefin polymer (COP)	2.325 ± 0.008	0.00053 ± 0.00004	2.289 ± 0.035	0.00027 ± 0.00005	2.307 ± 0.021	0.00040 ± 0.000045
Polycarbonate (PC)	2.765 ± 0.005	0.0057 ± 0.0002	2.754 ± 0.013	0.0054 ± 0.0007	2.759 ± 0.009	0.0055 ± 0.0004

**Table 4:** Empty resonator (R_A_ and R_B_) parameters (units GHz, mm, S/m, **°**C) at different temperatures.

R_A_ (TE_011_)		R_B_ (TM_010_)		Temperature (°C)
*f_e*1*_/Q_e*1*_*	D*_eqv*1*_*/*σ_eqv*1*_*	*f_e*2*_/Q_e*2*_*	D*_eqv*2*_/σ_eqv*2*_*	
13.1659/16,090	30.0182/2.21 × 10^7^	7.6540/7080	29.9822/3.69 × 10^7^	−40
13.1638/15,555	30.0238/2.06 × 10^7^	7.6503/6890	29.9969/3.49 × 10^7^	−20
13.1587/15,490	30.0373/2.05 × 10^7^	7.6492/6800	30.0013/3.41 × 10^7^	0
13.1555/14,950	30.0457/1.91 × 10^7^	7.6469/6573	30.0104/3.18 × 10^7^	+20
13.1503/14,730	30.0598/1.85 × 10^7^	7.6448/6540	30.0186/3.15 × 10^7^	+40
13.1448/14,430	30.0743/1.78 × 10^7^	7.6432/6410	30.0257/3.11 × 10^7^	+70
13.1393/14,140	30.0786/1.70 × 10^7^	7.6417/6378	30.0332/3.08 × 10^7^	+80
13.1393/13,920	30.0830/1.62 × 10^7^	7.6405/6343	30.0417/3.02 × 10^7^	+90
13.1290/13,615	30.0910/1.59 × 10^7^	7.6389/6305	30.0506/2.96 × 10^7^	+100
13.1235/13,285	30.0998/1.51 × 10^7^	7.6375/6275	30.0602/2.91 × 10^7^	+110

**Table 5:** Measured values of dielectric parameters of PDMS at different temperatures (parallel values—12.5 GHz; perpendicular values—7.5 GHz; equivalent values—10 GHz).

*ε_par_*	tan*δ_par_*	*ε_perp_*	tan*δ_perp_*	*ε_eqv_*	tan*δ_eqv_*	Anisotropy (%) *Aniso_ε_/Aniso_tanδ_*	Temp. (°C)
2.807	0.0234	2.791	0.0270	2.802	0.0252	0.7/−14	–40
2.782	0.0208	2.699	0.0243	2.744	0.0232	3.3/−18	–20
2.737	0.0192	2.642	0.0223	2.688	0.0214	3.8/−13	0
2.715 ± 0.011	0.0216 ± 0.007	2.592 ± 0.02	0.0184 ± 0.009	2.663 ± 0.03	0.0192 ± 0.009	4.7/15	+20
2.622	0.0209	2.611	0.0162	2.588	0.0177	0.3/28	+40
2.545	0.0192	2.573	0.0163	2.553	0.0162	1.2/16	+70

## Data Availability

Not applicable.

## References

[1] Yin A., Zhang C., Luo J., Liu J., Ren Z., Wang Y., Ye Y., Yin R., Feng Q., Chen Y. (2023). A highly sensitive and miniaturized wearable antenna based on MXene films for strain sensing. Mater. Adv..

[2] Najafi Khoshnoo S., Kim T., Tavares-Negrete J.A., Pei X., Das P., Lee S.W., Rajendran J., Esfandyarpour R. (2023). A 3D Nanomaterials-Printed Wearable, Battery-Free, Biocompatible, Flexible, and Wireless pH Sensor System for Real-Time Health Monitoring. Adv. Mater. Technol..

[3] Shah A.H., Patel P.N. (2023). Embroidered Annular Elliptical E-Textile Antenna Sensor for Knee Effusion Diagnosis. IEEE Sens. J..

[4] Pal A., Ahmad D., Pal S., Ghazali A.N. (2023). Efficient and low SAR dual functional wearable antenna in RFID ISM and GPS L1 bands for positioning applications. Wirel. Netw..

[5] Anilkumar T., Madhav B.T., Rao M.V., Nadh B.P., Kumar P.R. (2023). Automotive communication applications based circular ring antenna with reconfigurability and conformal nature. Int. J. Commun. Syst..

[6] Conti S., Nepa F., Di Pascoli S., Brunetti I., Pimpolari L., Song X., Parvez K., Javanbakht Lomeri H., De Rossi F., Lucarelli G. (2023). Hybrid flexible NFC sensor on paper. IEEE J. Flex. Electron..

[7] Ou X., Chen P., Liu B.-F. (2023). Optical Technologies for Single-Cell Analysis on Microchips. Chemosensors.

[8] Ren L.-F., Xia F., Shao J., Zhang X., Li J. (2017). Experimental investigation of the effect of electrospinning parameters on properties of superhydrophobic PDMS/PMMA membrane and its application in membrane distillation. Desalination.

[9] Miranda I., Souza A., Sousa P., Ribeiro J., Castanheira E.M., Lima R., Minas G. (2021). Properties and applications of PDMS for biomedical engineering: A review. J. Funct. Biomater..

[10] Mukesh A.N., Sharma P.K., Yadav V.P., Payal P.O., Solanki L. Design and Analysis of an Edge Truncated Flexible Antenna for Wi-Fi Applications. Proceedings of the 2022 International Conference on Electronics and Renewable Systems (ICEARS).

[11] Sharma P.K., Gupta N. (2022). A CPW-fed circular SRR-inspired flexible antenna using polydimethylsiloxane (PDMS) substrate for WLAN and WBAN applications. IEEE J. Flex. Electron..

[12] Simorangkir R.B.V.B., Kiourti A., Esselle K.P. (2018). UWB wearable antenna with a full ground plane based on PDMS-embedded conductive fabric. IEEE Antennas Wirel. Propag. Lett..

[13] Abbas S.M., Desai S.C., Esselle K.P., Volakis J.L., Hashmi R.M. (2018). Design and characterization of a flexible wideband antenna using polydimethylsiloxane composite substrate. Int. J. Antennas Propag..

[14] Janapala D.K., Nesasudha M., Neebha T.M., Kumar R. (2022). Design and development of flexible PDMS antenna for UWB-WBAN applications. Wirel. Pers. Commun..

[15] Kent G. (1996). Nondestructive permittivity measurement of substrates. IEEE Trans. Instrum. Meas..

[16] Hasar U.C., Kaya Y., Ozturk H., Izginli M., Ertugrul M., Barroso J.J., Ramahi O.M. (2022). Improved Method for Permittivity Determination of Dielectric Samples by Free-Space Measurements. IEEE Trans. Instrum. Meas..

[17] Courtney W.E. (1970). Analysis and Evaluation of a Method of Measuring the Complex Permittivity and Permeability Microwave Insulators. IEEE Trans. Microw. Theory Tech..

[18] Kato Y., Horibe M. Broadband Permittivity Measurements Using a Frequency-Variable Balanced-Type Circular-Disk Resonator. Proceedings of the 2018 Conference on Precision Electromagnetic Measurements (CPEM 2018).

[19] Dankov P.I. (2006). Two-resonator method for measurement of dielectric anisotropy in multilayer samples. IEEE Trans. Microw. Theory Tech..

[20] Gouda F., Anderson G., Matuszczyk M., Matuszczyk T., Skarp K., Lagerwall S.T. (1990). Dielectric anisotropy and dielectric torque in ferroelectric liquid crystals and their importance for electro-optic device performance. J. Appl. Phys..

[21] Zhang G., Sun Y., Qian B., Gao H., Zuo D. (2020). Experimental study on mechanical performance of polydimethylsiloxane (PDMS) at various temperatures. Polym. Test..

[22] Ali Khan M.U., Raad R., Tubbal F., Theoharis P.I., Liu S., Foroughi J. (2011). Bending analysis of polymer-based flexible antennas for wearable, general IoT applications: A review. Polymers.

